# The Adaptive Significance of Enamel Loss in the Mandibular Incisors of Cercopithecine Primates (Mammalia: Cercopithecidae): A Finite Element Modelling Study

**DOI:** 10.1371/journal.pone.0097677

**Published:** 2014-05-15

**Authors:** Kornelius Kupczik, Netta Lev-Tov Chattah

**Affiliations:** 1 Max Planck Weizmann Center for Integrative Archaeology and Anthropology, Max Planck Institute for Evolutionary Anthropology, Leipzig, Germany; 2 Institut für Spezielle Zoologie und Evolutionsbiologie, Friedrich-Schiller-Universität Jena, Jena, Germany; 3 The Department of Identification and Forensic Science, Israel National Police, Jerusalem, Israel; Museo Nazionale Preistorico Etnografico 'L. Pigorini', Italy

## Abstract

In several primate groups enamel is reduced or absent from the lingual (tongue) side of the mandibular incisor crowns akin to other placental and marsupial mammalian groups such as rodents, lagomorphs and wombats. Here we investigate the presumed adaptation of crowns with unilateral enamel to the incision of tough foods in cercopithecines, an Old World monkey subfamily, using a simulation approach. We developed and validated a finite element model of the lower central incisor of the rhesus macaque (*Macaca mulatta*) with labial enamel only to compute three-dimensional displacements and maximum principal stresses on the crown subjected to compressive loads varying in orientation. Moreover, we developed a model of a macaque incisor with enamel present on both labial and lingual aspects, thus resembling the ancestral condition found in the sister taxon, the leaf-eating colobines. The results showed that, concomitant with experimental results, the cercopithecine crown with unilateral enamel bends predominantly towards the inside of the mouth, while displacements decreased when both labial and lingual enamel are present. Importantly, the cercopithecine incisor crown experienced lower maximum principal stress on the lingual side compared to the incisor with enamel on the lingual and labial aspects under non-axial loads directed either towards the inside or outside of the mouth. These findings suggest that cercopithecine mandibular incisors are adapted to a wide range of ingestive behaviours compared to colobines. We conclude that the evolutionary loss of lingual enamel in cercopithecines has conferred a safeguard against crown failure under a loading regime assumed for the ingestion (peeling, scraping) of tough-skinned fruits.

## Introduction

The teeth of mammals have adopted a wide range of shapes and sizes which are assumed to be specifically suited for their designated functions; ingestion and mastication. Experimental and numerical studies have underlined the importance of the enamel component in determining the biomechanical response of mammalian teeth under load [1]–[9]. These studies show that the gross external and internal morphology and the structure of the highly stiff and brittle enamel overlaying the softer and tougher dentine core is a primary factor in governing the mode of deformation in different primate and carnivoran species. The importance of the crown shape in the response of teeth to load leads back to the question of how specific morphological features have evolved to adapt to certain feeding functions. Here, we approach the above issue by using experimentally validated numerical simulations to investigate the mechanical behaviour of the derived crown morphology of the mandibular incisors of cercopithecine primates, one of the Old World monkey subfamilies. In this primate group, unlike in the maxillary incisor crown with enamel on both the labial and lingual side, the enamel of the mandibular incisors is restricted to one side of the crown only. We are particularly interested in the question whether this peculiar dental morphology constitutes an adaptive advantage over having enamel on both sides of the crown.

**Figure 1:**
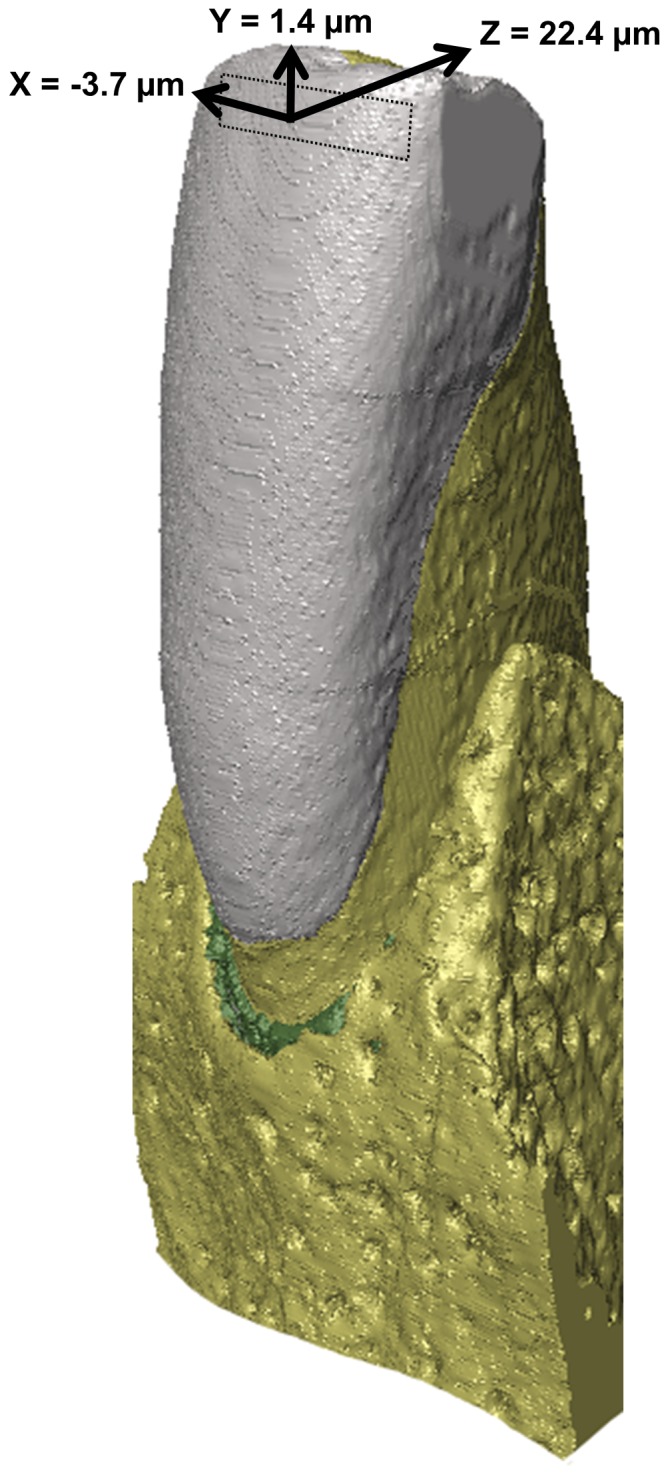
Displacements in a mandibular central incisor of a macaque under an axial load (F). X, Y and Z indicate displacements in the three orthogonal planes (data from Lev-Tov Chattah et al. [7], their Fig. 2).

In the mandibular incisors of the fruit and seed dependent cercopithecines (macaques, baboons and vervet monkeys) and tree-gouging primates such as the callitrichids and the strepsirhine *Daubentonia*, the lingual enamel is missing creating an enamel morphology which mostly resembles a curved plate overlaying the dentine core [10]–[13], although apparently an extremely thin enamel layer was found in a *Macaca fuscata* lower incisor [14]. Unlike cercopithecines, the ancestral condition of anthropoid primates is found in the sister taxon, the (predominantly) leaf-eating colobines, and in hominoids, where a significant layer of enamel is present both on the labial and lingual crown surface [10], [15]. In hominoids the thickness of the lingual enamel is about two thirds that of the labial enamel [16], [17], while in colobines it is less than a quarter (17–21%; [12]). It has been argued that the difference in lower incisor enamel cap morphology between cercopithecines and colobines is related to different dietary specializations and ingestion techniques [12], [18]. In colobines, observed modes of ingestion are nipping, incising and stripping off leaves by dragging a branch between their mandibular and maxillary incisors with their hands [19]–[22]. Here, the main function of the incisors is to provide grip with relatively little force involved [12]. In contrast, cercopithecines often use their incisors to incise and scrape the skin of fruits where the labial surface of the mandibular incisor crown scrapes across the hard surface of a seed within the fruit [19], [20], [23]. Thus, the presence of enamel on the labial aspect and its absence on the lingual aspect in cercopithecines has the particular advantage of maintaining a sharp edge at the enamel-dentine boundary which is essential for effective incision of tough fruit pericarp [10], [12]. The orientation of the load at which the incisor crown touches the food item during the incision mode can be assumed to be parallel or near parallel to the long axis of the incisor (i.e. axial), while the load orientation of the scraping mode will be non-axial (see Figure 5 in [23]). Lev-Tov Chattah et al. [7] showed experimentally in mandibular incisors of the rhesus macaque (*Macaca mulatta*) that the lack of lingual enamel confers a deformation mode to the extent that the crown predominantly deflects in the labio-lingual direction and less so in the superior-inferior direction under a compressive load parallel to the long axis of the tooth (Fig. 1). This mechanical behaviour differs markedly from the one found in crowns with a full enamel cap such as premolars and molars [2], [24].

**Figure 2:**
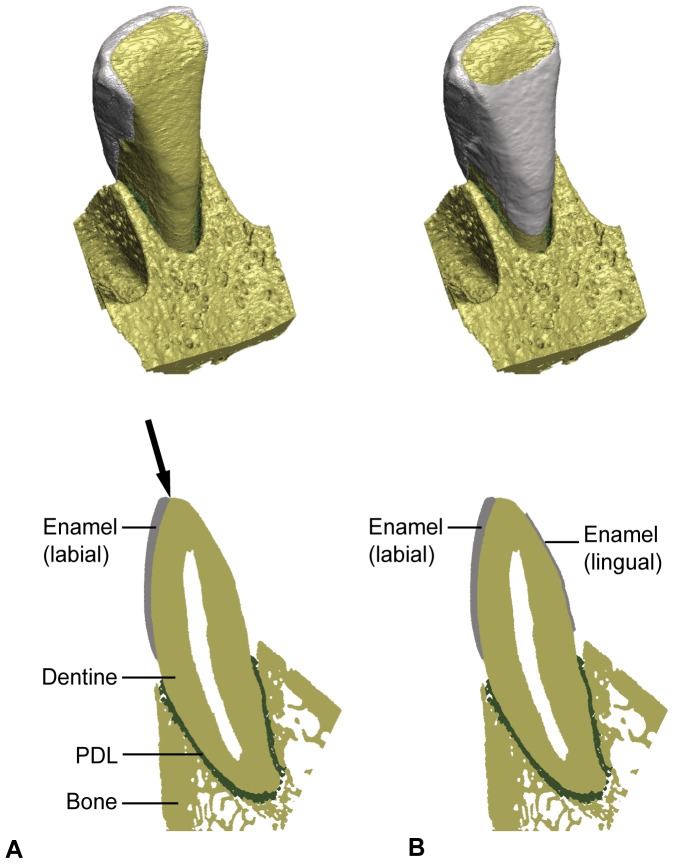
3D models of macaque mandibular central incisor with labial enamel (A, top row) and both labial and lingual enamel (B, top row). The bottom row shows longitudinal sections through the models and illustrates the dental materials included in analysis. Labial is to the left. PDL  =  periodontal ligament.

In order to investigate the functional significance of the derived incisor morphology in cercopithecines, in the present paper we developed a finite element (FE) model of the lower central incisor of *M. mulatta* and validated it against our previously published experimental results [7]. The advantage of this simulation approach is that it enables an *in silico* analysis of the three-dimensional mechanical behaviour of both natural and hypothetical crown morphologies under load. Thus, we compared the deformation pattern of the FE model of the incisor crown with labial enamel only to that resulting from an FE model with both labial and lingual enamel. The latter hence mimicked the ancestral situation of Old World monkeys present in colobines. We hypothesised that by adding lingual enamel the crown would bend less in the labio-lingual direction under an axial compressive load reflecting the scraping ingestion mode (cf. Fig. 1). In comparison, we also analysed the stress distributions in both models for a range of non-axial loads including the one assumed for a leaf-stripping ingestion mode. This approach enabled us to better understand the complex issue of dental form-function relationships and to relate them to dietary adaptations in both cercopithecine and colobine primates.

## Materials and Methods

### Ethics statement

We used micro-computed tomography (µCT) scans of the mandible of a male *M. mulatta* cadaver (specimen number DPZ 7845) acquired for an earlier study (see [7]). This specimen was obtained post mortem from the German Primate Center (DPZ, Göttingen, Germany). The animal was sacrificed following an experiment unrelated to either the present or the previous study. The animal was kept under the regulations for non-human primates by the guidelines for the accommodation and care of animals used for experimental and other scientific purposes (2007/526/EC; Appendix A ETS 123), and experiments and procedures were performed in accordance with the regulations of the German Animal Welfare Act.

### Computed tomography and image segmentation

The mandible was scanned at 28 µm resolution with a BIR ACTIS 225 / 300 high-resolution industrial µCT scanner (Department of Human Evolution, Max Planck Institute for Evolutionary Anthropology, Leipzig). In Avizo 6.3 (Visualization Sciences Group) the dataset was resampled to 56 µm voxel size and cropped to capture the mandibular right central incisor and a portion of the bony symphysis housing the alveolar socket with the tooth root. The tip of the incisor was moderately worn resulting in a flat incisal plane formed by the enamel and dentine (Fig. 2). We segmented the enamel, dentine, periodontal ligament (PDL) and bone (no distinction was made between cortical and trabecular bone) using a threshold based approach in Avizo. The PDL was segmented as the space between the root dentine and the alveolar bone socket (Fig. 2A). The pulp cavity was not segmented and the tooth was thus left hollow (i.e. air filled). Moreover, it was not possible to discern cementum from dentine. This model was called the labial model (LAB) and served as the reference model for subsequent analyses. Dental tissue volumes and the enamel thickness are given in Table 1. Enamel thickness is reported for the sagittal (bucco-lingual) plane measured at right angles below the incisal margin following Shellis and Hiiemae [12].

**Table 1:** Enamel thickness (in mm), tissue volumes (in mm^3^) and elastic material properties in five FE models.

	LAB	LING	Young's modulus/Poisson's ratio^1^
Enamel thickness^2^ (labial/lingual)	0.56/-	0.56/0.15	-
Enamel volume	20.25	25.11	95/0.3
Dentine volume	143.45	143.45	25/0.23
PDL volume	22.45	22.45	0.05/0.49
Bone volume	432.53	432.53	95/0.3

1 Young's modulus in GPa; Poisson's ratio is unitless.

2 thickness measured at right angles below incisal margin.

In addition, we produced a model in which we added voxels on the lingual side of the crown overlaying the dentine (model LING). This lingual enamel extended from below the incisal plane to a few millimetres above the alveolar margin (Fig. 2B). The thickness and volume of the lingual enamel was 27% and 24%, respectively, of that of the labial enamel. This ratio matches that found in colobine monkeys [12].

### Finite element modelling

The segmented images of both models (LAB, LING) were exported as a stack of bitmap images and the dataset was converted into a finite element (FE) mesh file consisting of 3,460,653 eight-noded cubic elements using custom software. The FE mesh files were imported into VOX-FE, a custom FE preprocessing software and solver [25], [26]. We then assigned isotropic, linear elastic properties (Young's modulus of elasticity and Poisson's ratio) to the materials involved based on literature values for human teeth [27]–[29] (Table 1). The periodontal ligament was treated as a bulk tissue and hence a single Young's modulus value was assigned. Since the FE solver used in this study is limited to analysing three different materials at any one time, we used the same elastic properties for both dentine and bone, albeit separated by the PDL. Both models can be made available upon request.

**Figure 3:**
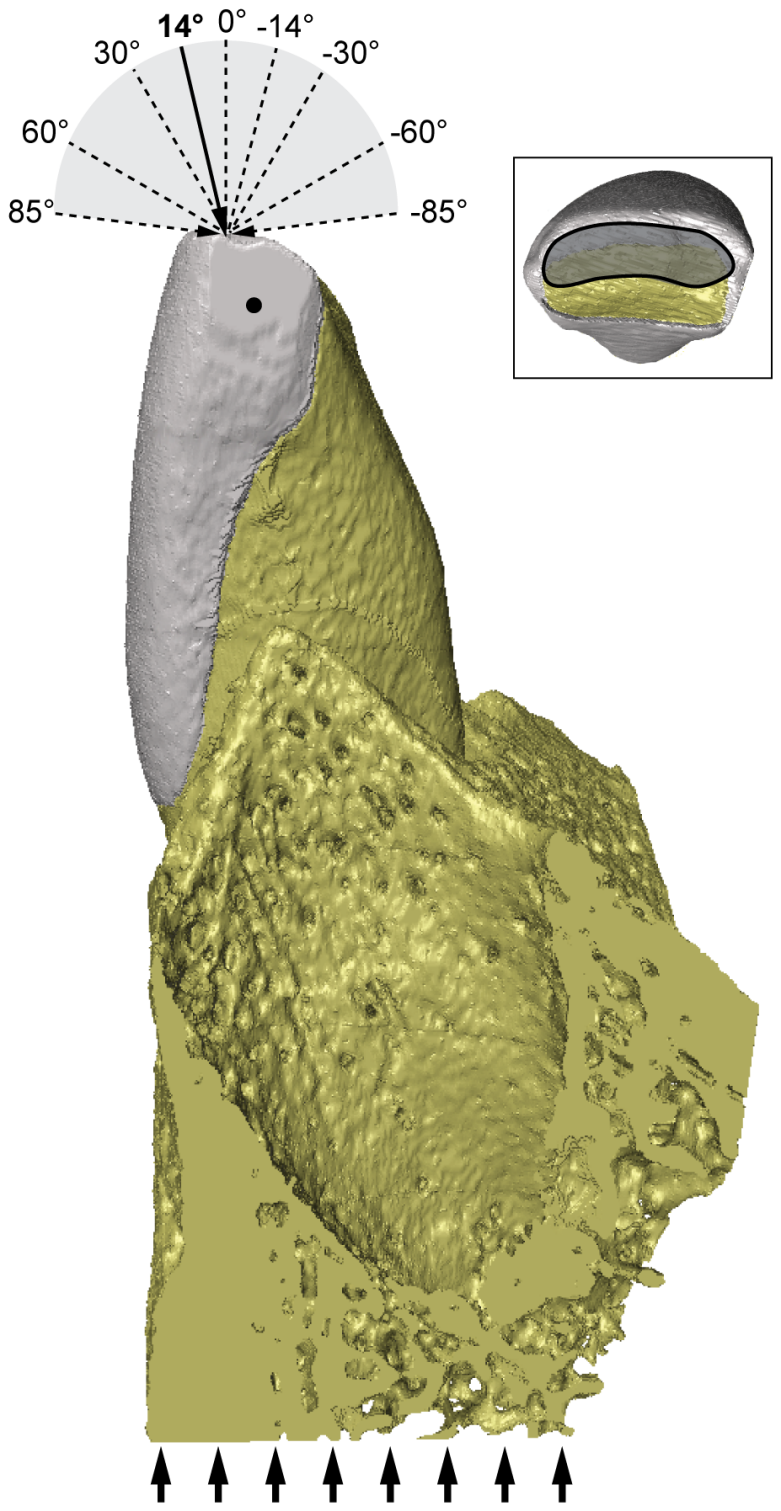
Lateral view of incisor model illustrating constraints (thick arrows and black dot) and loads applied (thin arrows) in FEA. Numbers indicate different load angles in the labio-lingual direction. A load angle of 14° is along the long axis of the tooth and corresponds to the experimental loading regime. Inset shows occlusal view of model and indicates area where forces were applied to.

Both models were constrained at the base of the symphyseal bone block with 425 nodes in x, y and z and on the mesial and distal sides of the incisor crown with one node each in the y-direction only (Fig. 3). A force of 72 N directed parallel to the long axis of the tooth was thus applied to 2396 surface nodes on the incisal area of the incisor (Figs. 2 and 3). The direction and magnitude of load corresponded to those used in Lev-Tov Chattah et al. (2011), and deviates 14° in the labio-lingual plane from an axis perpendicular to the base of the model (Fig. 3). This load direction corresponds to the scraping ingestion mode assumed for fruit eating cercopithecines following Ungar [19] and Agrawal et al. [23]. In addition, we assessed the sensitivity of the LAB and LING models to variations in load direction ranging from +85° to −85° in the labio-lingual plane (see Fig. 3). A load of 0° was parallel to the labial enamel, while an angle of +85° implied a lingually directed force parallel to the incisal plane and an angle of −85° was towards the outside of the mouth. The latter thus corresponded to the leaf-stripping ingestion mode.

VOX-FE was used to solve the voxel-based models and to compute x,y,z-displacements, total displacement (both in µm) and maximum principal stress (in MPa). Nodal displacements along the x-axis are equivalent to the medio-lateral direction; y-displacements are equivalent to the superior-inferior direction and z-displacements correspond to the labio-lingual direction (cf. Fig. 1). Maximum principal (i.e. tensile) stress was chosen because it is a commonly used failure criterion for brittle materials such as enamel [3], [30]–[33]. To eliminate artefacts on the model surface resulting from the use of brick elements, the maximum principal stress maps were smoothed prior to analysis. The mechanical parameters were extracted from surface elements at four locations on the tooth model (superior-labial, inferior-labial, superior-lingual, inferior-lingual). For validation, we compared the displacement results of the LAB model at the superior-labial location with those derived from a study using electronic speckle pattern interferometry (ESPI) where x,y,z displacements were measured on the labial enamel surface of the central incisor in the mandible [7] The load was exerted parallel to the long axis of the tooth in labio-lingual orientation (14°; Fig. 3). It was found that the displacements were highest in the z-direction towards the inside of the mouth followed by x-displacements towards the right side and the least displacements occurred upwards in the y-direction ([7]; experimental x,y,z displacements are given in Fig. 1). The total displacement was 22.75 µm.

## Results

The FE analysis of the LAB model yielded a total displacement value at the superior-labial location almost identical to the experimentally derived result; the difference was 0.1% (Table 2; Fig. 1). In both the experiment and the model the largest displacement was in the z-direction towards the lingual side of the tooth. In contrast, the FE analysis resulted in smaller x-displacements (towards the mesial side) and larger y-displacements (upwards towards the incisal edge) compared to the experiment (81% and 120% difference, respectively). Despite this difference the deformation pattern of the incisor crown *in vitro* and *in silico* is similar overall and thus the model can be regarded as validated.

**Table 2:** Mean and standard deviation of nodal displacements (x, y, z and total) derived from FE models of mandibular central incisor with labial enamel only (LAB) and labial lingual enamel (LING) under a load along the long axis of the tooth.

	LAB				LING				Δ % LAB/LING^e^			
	X	Y	Z	Total	X	Y	Z	Total	X	Y	Z	Total
Sup lab^a^	−0.7±0.1	3.1±0.6	22.5±0.4	22.7±0.3	−0.5±0.1	3.2±0.4	18.3±0.3	18.6±0.2	−31	2	−18	−18
Inf lab^b^	0.4±0.0	1.8±0.2	11.9±0.2	12.0±0.2	0.4±0.0	2.0±0.1	9.9±0.1	10.1±0.1	−3	9	−17	−16
Sup ling^c^	−0.04±0.1	7.9±0.2	21.7±0.2	23.1±0.1	0.03±0.1	7.0±0.2	17.7±0.2	19.1±0.1	−169	−11	−18	−18
Inf ling^d^	0.9±0.0	9.3±0.2	16.0±0.1	18.5±0.2	0.7±0.0	8.4±0.2	13.4±0.1	15.8±0.2	−13	−10	−17	−15

Displacements are in µm.

a Average and standard deviation of 699 nodes.

b Average and standard deviation of 247 nodes.

c Average and standard deviation of 361 nodes.

d Average and standard deviation of 549 nodes.

e Percentage difference between models LAB and LING.

When the models with and without lingual enamel are compared, the overall pattern of x, y, z displacements was found to be the same, i.e. the largest displacement was in the z-direction and the smallest in the x-direction (Table 2). As expected, the displacements in the z-direction were consistently less in model LING sampled at the four locations, i.e. the deflection of the crown towards the lingual side was reduced when lingual enamel is present. Compared to the LAB model displacements in the y-direction increased to some extent on the labial enamel but decreased on the lingual enamel surface. Displacements in the x-direction varied more strongly between the LAB and LING models. While the top of the crown deformed towards the mesial side (note the negative values at the two superior locations) and the inferior part deformed to the distal side, only the superior labial location tilted towards the mesial side in model LING (Table 2).

**Figure 4:**
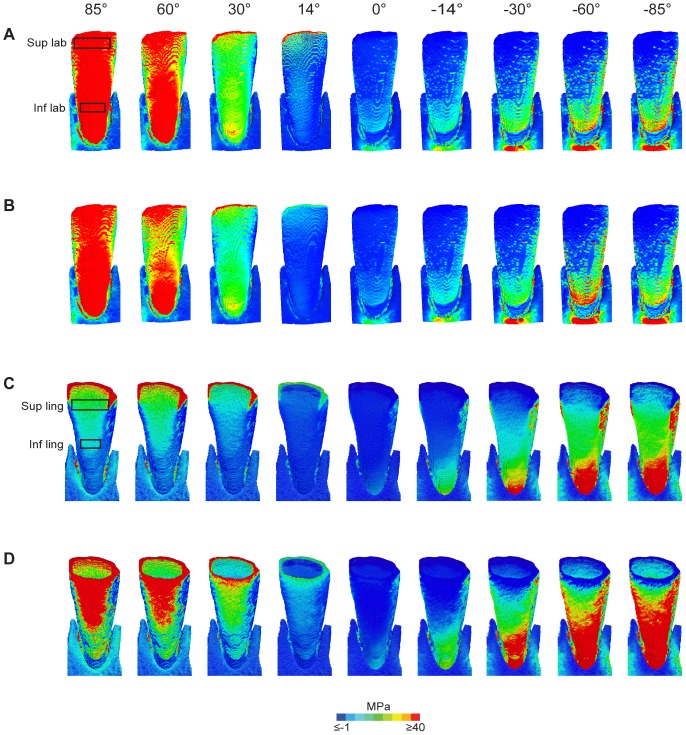
Maximum principal stress (MPa) maps in model with labial enamel (A, C) and model with both labial and lingual enamel (B, D) under different loading conditions. A and B are labial views, and C and D lingual views. Boxes show locations on labial (superior and inferior) and lingual surfaces (superior and inferior) from which means were calculated. Sup lab  =  superior labial; Inf lab  =  inferior labial; Sup ling  =  superior lingual; Inf ling  =  inferior lingual.

**Figure 5:**
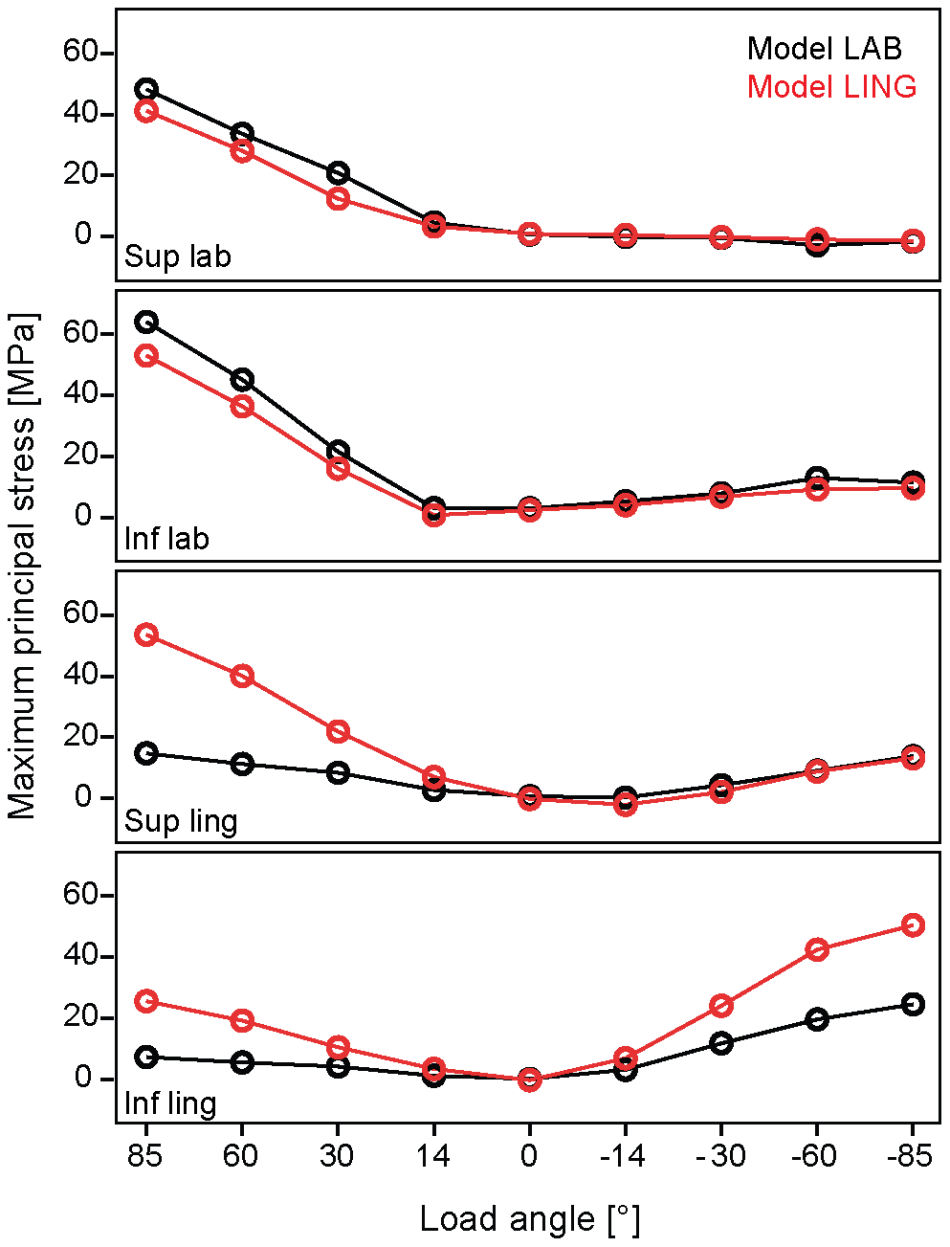
Maximum principal stress (MPa) mean values at four locations on labial and lingual surface of incisor in model with labial enamel (LAB) and in model with labial and lingual enamel (LING). Same abbreviation as in Fig. 4.

When maximum principal stresses are considered, in model LAB the values were higher on the labial aspect than on the lingual aspect under a load parallel to the tooth's long axis (14°) (Table S1; Fig. 4). Maximum principal stresses increased the more the load was applied towards the inside of the mouth (positive load angles) with particularly high values at the inferior labial location (Fig. 5). When the crown was subjected to a 0° load, i.e. parallel to the long axis of the labial enamel, maximum principal stress values were lowest of all loading conditions (Fig. 4). Non-axial loadings towards the outside of the mouth (i.e. negative load angles) resulted in an increase of maximum principal stresses particularly in the inferior lingual location (Fig. 4).

In the LING model under the load regimes ranging from 14° to 85° maximum principal stresses were slightly lower on the labial side but higher on the lingual side compared to model LAB (Table S1; Fig. 5). When subjected to a 0° loading the crown experienced very low tensile and compressive stresses. In comparison with model LAB the labially oriented loading regimes (negative angles) in model LING yielded somewhat lower maximum principal stresses on the labial enamel and at the superior-lingual location but high values at the inferior lingual location (Fig. 4, 5).

## Discussion

It was shown previously that under non-physiological, axial load the central mandibular incisors of *M. mulatta* deformed very little in compression in the superio-inferior direction but that it rather deformed predominantly towards the oral cavity [7]. This mechanical behaviour was different to that observed in fully capped premolar and molar crowns of humans and minipigs [2], [9], [24], and it was mainly attributed to the lack of lingual enamel and the gross morphology of the cercopithecine incisor crown [7]. By using a simulation approach as an extension of the experimental study the main hypothesis assessed in the present study is supported insofar as the deformation pattern indeed changes when lingual enamel is included in the model. As expected, the crown becomes stiffer overall and deflects less in the lingual direction (by up to 18%) (Table 2). It thus approaches the deformation pattern observed in fully capped postcanine tooth crowns.

While there were relatively high absolute differences between the x and y displacements derived experimentally and through the simulations (model LAB), there was only a negligible difference for the z displacement and the total displacement. Crucially, the FE analyses yielded similar directions of deformation, i.e. the largest displacements occurred towards the oral cavity (z-axis) which agrees with the experimental results [7]. Although it can only be speculated at this point, the model constraints (see Fig. 3) and the simplification of the material properties of the mineralised and soft tissues modelled here as homogeneous and isotropic may account for the absolute differences in displacements. It is well known that both enamel and dentine have heterogeneous and orthotropic Young's moduli [34]–[37], and thus more realistic elastic properties should ideally be taken into account in future models.

**Figure 6:**
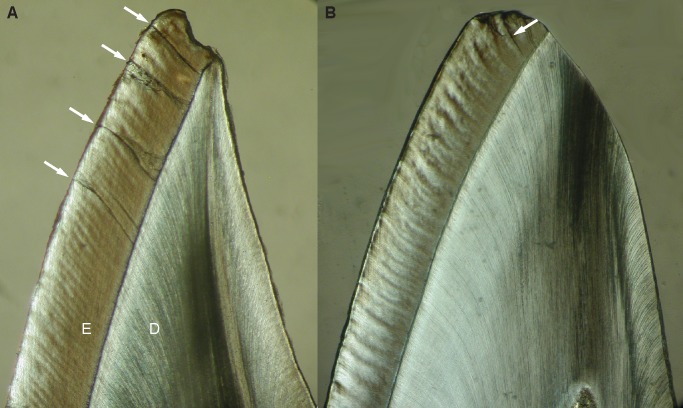
Longitudinal sections through an unworn (A) and a worn (B) mandibular central incisor crown of *Macaca mulatta* (collection of Peter Shellis). The arrows indicate cracks in the enamel (E) which run parallel to the dark and light Hunter-Schreger bands from the enamel-dentine junction to the outer enamel surface. Note the orientation of the crack in B) which is near parallel to the worn incisal plane. D  =  dentine. Images not to scale. Photos courtesy of Chris Dean.

From a tooth failure point of view, maximum principal stress is the crucial criterion for the mechanical behaviour of brittle materials [3], [5], [32]. When loads are applied along the long axis of the labial enamel cap (0°) or along the long axis of the tooth (14°) the crown is subjected to low tensile or even compressive stresses both when lingual enamel is present or absent. In contrast, loads directed towards the inside of the mouth (i.e. load angles ≥ 30°), which are assumed to occur during the scraping ingestion mode of cercopithecine primates, may yield critically high stresses on the crown, in particular on the labial aspect. This type of ingestion implies that the hard seed of a fruit pushes against the labial aspect of the mandibular incisor crown (cf. [23]) thus bending the curved labial enamel in the labio-lingual direction and resulting in high tensile stress. It is well established that enamel better resists compression than tension as reflected by a higher ultimate compressive strength than ultimate tensile strength in human mandibular molar enamel [3], [8], [17], [38]. Tensile induced enamel fractures occur along (i.e. parallel to) the axis of the prisms [39], and this can be observed in longitudinal sections of macaque mandibular incisor crowns (Fig. 6). During ingestion the enamel is thus shed parallel to the incisal plane. In cercopithecines, the curved incisors both in the cervico-incisal and mesio-distal planes have been considered an adaptation to resisting labio-lingual bending stress when fracturing the (hard) pericarp of fruits [38], [39] (see Fig. 2). Observed chippings of incisors and labiolingually oriented (i.e. perpendicular to the incisal edge) striations on the labial surfaces of cercopithecine incisors support the notion that ingestion in this group of primates involves the application of high loads [22]. Given the simulation data presented here it is likely that labially directed loads as implied when incising and scraping off fruit pericarp lead to mechanical overloading of the incisors.

In the hypothetical case that enamel would be present on both sides of the cercopithecine crown (as is found in colobines and hominoids) tensile stress in the crown may also reach critically high stress levels on the lingual aspect of the crown (Table S1; Fig. 4, 5). This is likely to do with a buckling of the lingual enamel in the mesio-distal plane when the tooth is bent lingually. Moreover, the leaf-stripping ingestion mode (i.e. negative load angles) incurs large tensile stresses on large portions of the lingual enamel in model LING (see Fig. 4, 5). Indeed, Magne et al. [8] noted in maxillary incisors of modern humans (where enamel is present on both sides of the crown) that enamel on the lingual side exhibited a high crack propensity due to elevated tensile stresses under a horizontal load directed towards the outside of the mouth (akin to our −85° load direction). In colobines, the sister group to cercopithecines, with their strongly proclined incisors with blunt and flattened incisal surfaces [12], [40] the presence of both labial and lingual enamel has been attributed to a predominantly leaf dependent diet. Here the anterior teeth are used for tearing and stripping leaves, a function which involves moving the hand downward and away from the mouth [19], [22], [23], [41], [42]. This ingestion mode which requires relatively low incisal force is facilitated by an underbite where the lower incisors protrude in front of the maxillary incisors [43]. It may therefore be deduced that the deformation pattern of colobine mandibular incisors is likely to be distinct from that of cercopithecines. It is noteworthy that the stem group cercopithecid, *Victoriapithecus macinnesi*, has also enamel on both the labial and lingual aspects of the mandibular incisor [44] despite the fact that cranial and postcanine dental features suggest a diet of fruits, tubers and seeds [45], [46]. That notwithstanding, our results suggest that the derived cercopithecine incisor enamel morphology allows for more complex ingestive behaviour with a wider range of mechanical resistant foods compared to that of colobines. In other words, the adaptive advantage of having lost lingual enamel as a derived condition in cercopithecines may be that damages to the crown in the form of cracks are avoided when feeding on high-modulus and/or large food objects. Although Neotropical capuchins, which bear enamel on either side of the mandibular incisor crown, also engage in scraping and incising ingestion modes (*Cebus olivaceus*) and hard object ingestion (*Sapajus apella*), the range of load orientations may be distinct from that assumed in cercopithecines [47], [48]. Whether this is the case requires further investigation.

The reduction of lingual enamel has occurred several times during the evolution of mammals. The mandibular incisors of cercopithecines thus resemble those of other primates (callitrichids and *Daubentonia*) as well as rodents, lagomorphs and wombats [11], [13], [49]–[52]. The combination of stiff enamel and the more compliant dentine results in a sharp incisal edge for effective cutting of food material. Despite the parallel in incisor crown structure, it is unlikely that this feature evolved entirely for this one adaptive reason in all groups. Rodents, lagomorphs, wombats and *Daubentonia* possess gnawing incisors which are continuously erupting and are adapted to highly abrasive foods. A fractured crown poses less of a risk because it will eventually be replaced. In contrast, cercopithecines and perhaps also the tree-gouging callitrichids are under stronger selective pressure to maintain efficient food ingestion because tooth failure would have severe consequences on nutrient intake.

## Conclusions

By solving a series of finite element models of a cercopithecine mandibular incisor we have demonstrated the effect of absence or presence of lingual enamel on tooth deformation. In the natural condition with no lingual enamel maximum principal stress levels were lower compared to the ancestral situation where enamel was present lingually as found in the sister taxon, the leaf-eating colobines. We therefore conclude that the evolutionary loss of lingual enamel in the incisors of cercopithecine primates has conferred a safeguard against crown failure under a loading regime assumed for the ingestion (peeling, scraping) of tough-skinned fruits.

## Supporting Information

Table S1 Mean elemental maximum principal stress (MPa) at four locations on incisor crown in models with labial (LAB) and labial and lingual (LING) enamel with varying load direction.
(DOCX)
