# Supplementary material for: The Adaptive Significance of Enamel Loss in the Mandibular Incisors of Cercopithecine Primates (Mammalia: Cercopithecidae): A Finite Element Modelling Study
Source: PLoS One. 2014 May 15;9(5):e97677. doi: 10.1371/journal.pone.0097677 (PMC4022739; doi:10.1371/journal.pone.0097677)
Supplement: Table S1 — Mean elemental maximum principal stress (MPa) at four locations on incisor crown in models with labial (LAB) and labial and lingual (LING) enamel with varying load direction. (DOCX) [file pone.0097677.s001.docx]

Table S1. Mean elemental maximum principal stress (MPa) at four locations on incisor crown in models with labial (LAB) and labial and lingual (LING) enamel with varying load direction.

|  | 85° | | 60° | | 30° | | 14° | | 0° | | -14° | | -30° | | -60° | | -85° | |
| --- | --- | --- | --- | --- | --- | --- | --- | --- | --- | --- | --- | --- | --- | --- | --- | --- | --- | --- |
| Location | LAB | LING | LAB | LING | LAB | LING | LAB | LING | LAB | LING | LAB | LING | LAB | LING | LAB | LING | LAB | LING |
| Sup lab^a^ | 48.22 | 41.16 | 33.65 | 28.04 | 20.79 | 12.40 | 4.59 | 3.34 | 0.70 | 0.90 | -0.09 | 0.52 | -0.44 | -0.17 | -2.84 | -0.95 | -1.68 | -1.38 |
| Inf lab^b^ | 64.07 | 53.04 | 45.14 | 36.39 | 21.51 | 16.00 | 3.07 | 0.78 | 2.99 | 2.40 | 5.33 | 4.03 | 7.86 | 6.82 | 12.94 | 9.22 | 11.41 | 9.61 |
| Sup ling^c^ | 14.77 | 53.61 | 11.26 | 40.11 | 8.41 | 21.90 | 2.78 | 7.00 | 0.73 | -0.18 | 0.32 | -2.08 | 4.37 | 2.07 | 9.14 | 8.83 | 13.82 | 13.18 |
| Inf ling^d^ | 7.30 | 25.58 | 5.53 | 19.20 | 4.19 | 10.50 | 1.19 | 3.44 | 0.12 | -0.28 | 3.13 | 6.90 | 11.77 | 24.10 | 19.63 | 42.46 | 24.58 | 50.47 |

^a^ Superior labial; average of 1635 elements; ^b^ inferior labial; average of 698 elements; ^c^ superior lingual; average of 734 elements; ^d^ inferior lingual; average of 325 elements
